# Supplementary material for: Cooperation between SS18-SSX1 and miR-214 in Synovial Sarcoma Development and Progression
Source: Cancers (Basel). 2020 Jan 30;12(2):324. doi: 10.3390/cancers12020324 (PMC7072427; doi:10.3390/cancers12020324)
Supplement: Supplementary file 1 [file cancers-12-00324-s001.zip › cancers-692050-Suppl-final/cancers-692050-Supplementary Materials-corrected proof2.docx]

Supplementary Materials

Cooperation between SS18-SSX1 and miR-214 in Synovial Sarcoma Development and Progression

Miwa Tanaka, Mizuki Homme, Yukari Yamazaki, Keisuke Ae, Seiichi Matsumoto, Subbaya Subramanian and Takuro Nakamura


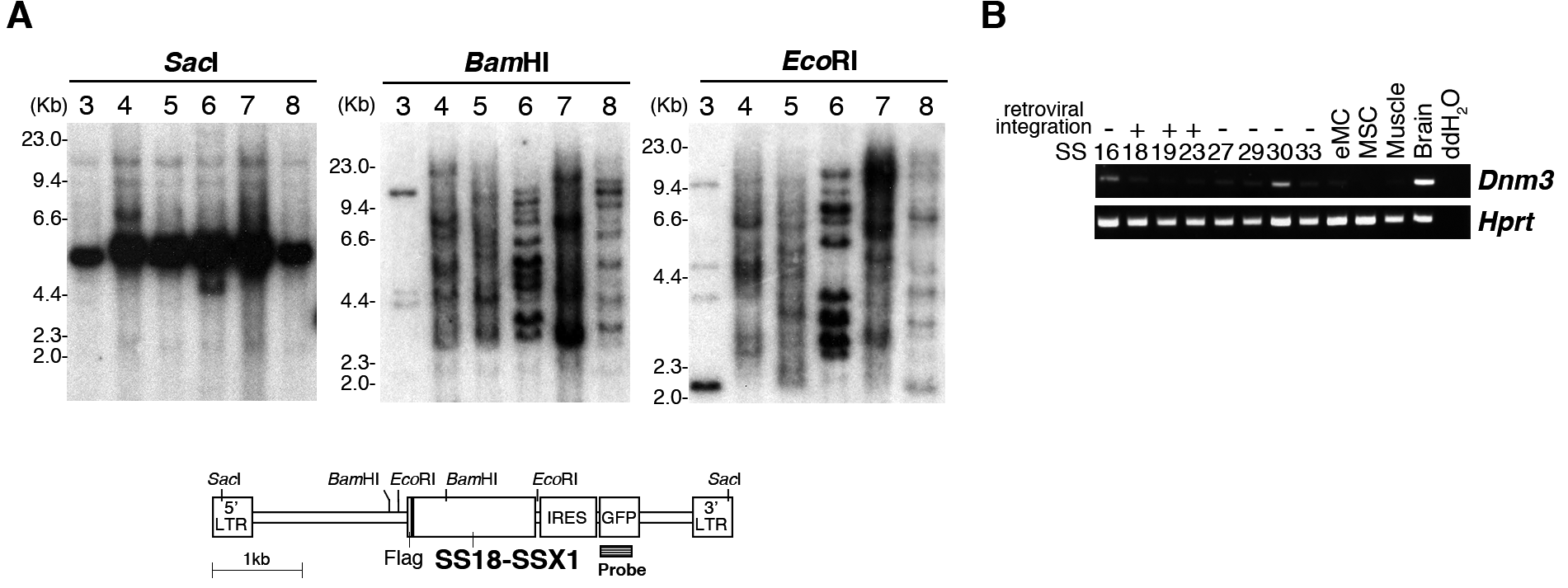


**Figure S1.** Retroviral integrations in mouse synovial sarcoma. (**A**) Southern blotting of synovial sarcoma tissue shows retroviral integrations of multiple copies. Restriction enzymes used for DNA digestion, restriction sites within the retroviral vector, and the probe are indicated. (**B**) RT-PCR of *Dnm3* in mouse synovial sarcoma. *Hprt* is used as a control.


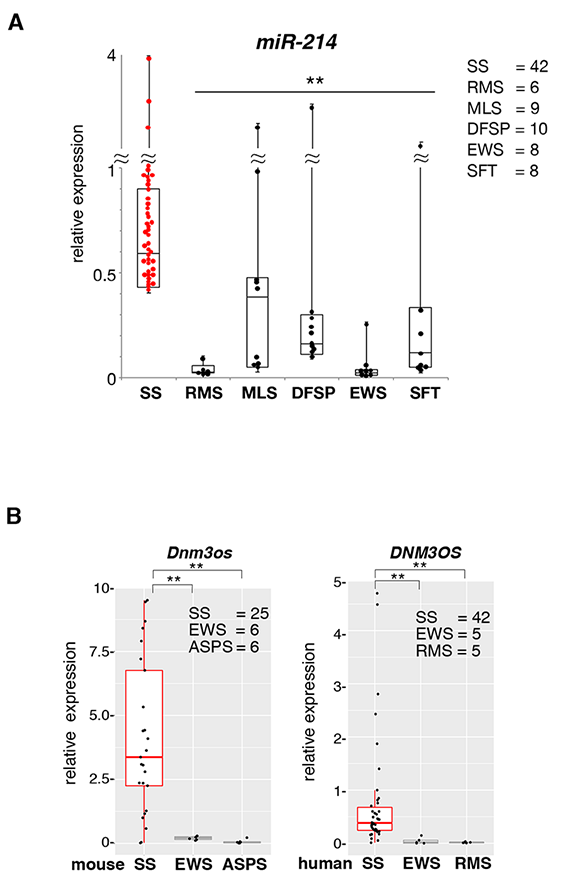


**Figure S2.** Upregulation of *miR-214* and *Dnm3os* in synovial sarcoma. (**A**) RT-qPCR for *miR-214* in human sarcoma tissues. SS: synovial sarcoma, RMS: rhabdomyosarcoma, MLS: myxoid liposarcoma, DFSP: dermatofibrosarcoma protuberans, EWS: Ewing sarcoma, SFT: solitary fibrous tumor. The numbers of cases examined are indicated. ** *p* < 0.01. (**B**) RT-qPCR for mouse *Dnm3os* (left) and human *DNM3OS* (right) in mouse and human sarcomas. SS: synovial sarcoma, EWS: Ewing sarcoma, ASPS: alveolar soft part sarcoma, RMS: rhabdomyosarcoma. The numbers of cases examined are indicated. ** *p* < 0.01.


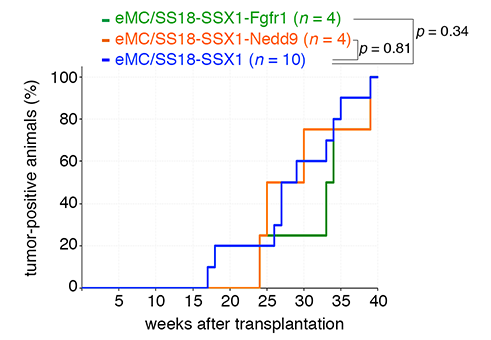


**Figure S3.** Cumulative incidences of synovial sarcoma with or without Fgfr1 or Nedd9. The number of mice used are indicated. Significance was examined by log-rank test.


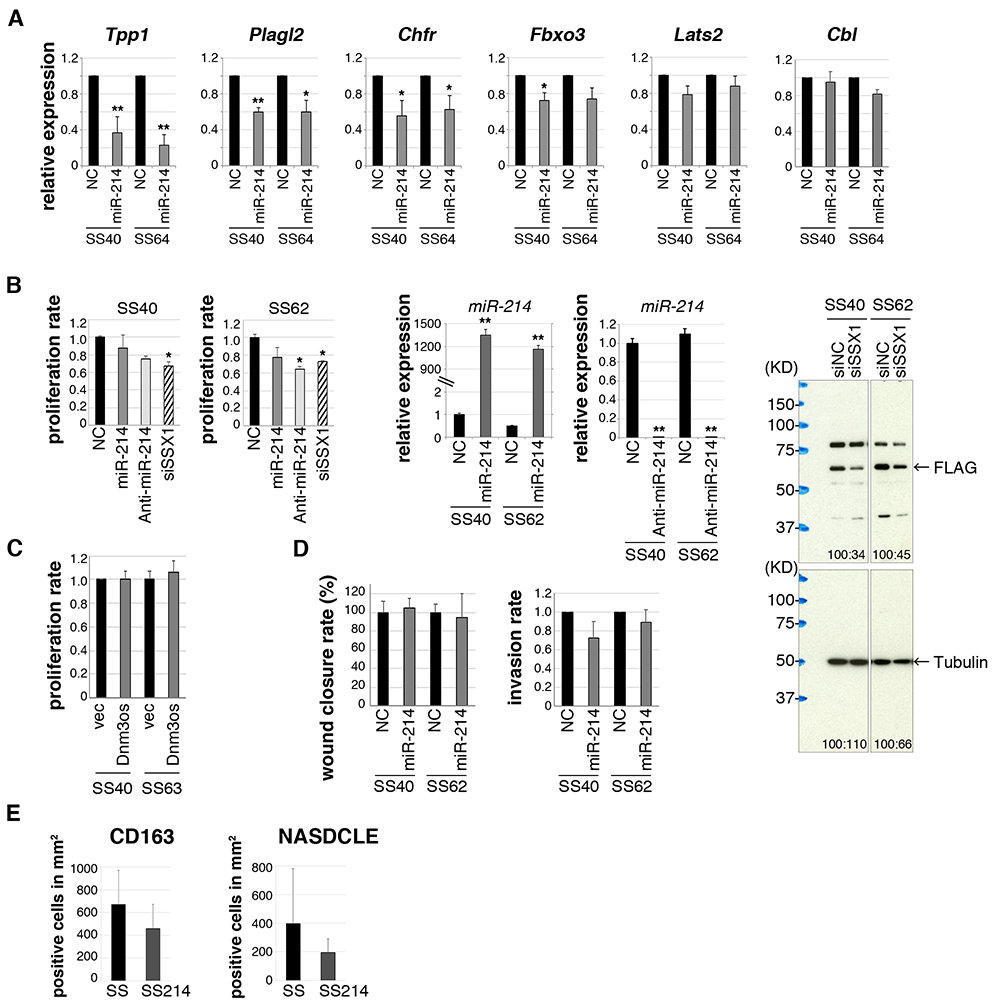


**Figure S4.** Effects of miR-214 expression in mouse synovial sarcoma. (**A**) RT-qPCR for candidate *miR-214* target genes, *Tpp1, Plagl2, Chfr, Fbxo3,* *Lats2,* and *Cbl* in synovial sarcoma cells with or without *miR-214*. * *P* < 0.05, ** *P* < 0.01. (**B**) Proliferation of synovial sarcoma cells with *miR-214, anti-miR-214* expression or *SS18-SSX1* knockdown (left). *miR-214* and SS18-SSX1 expression is confirmed by RT-qPCR or immunoblotting (right) * *P* < 0.05, ** *P* < 0.01. (**C**) Proliferation of synovial sarcoma cells with or without *Dnm3os* expression. (**D**) Migration and invasion activities of synovial sarcoma cells with or without *miR-214* were tested by wound healing assay (left) and invasion assay (right). (**E**) The number of CD163-positive macrophages and naphthol AS-D chloroacetate esterase (NASDCLE)-positive granulocytes in synovial sarcoma with (SS214, n = 10) or without miR-214 (SS, n = 11).


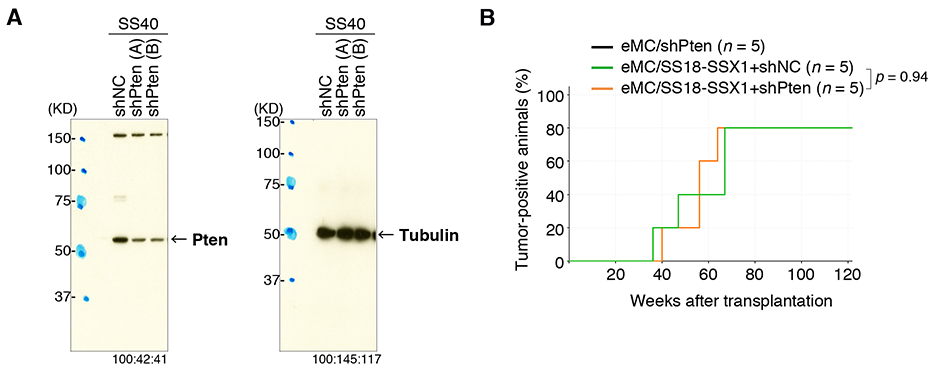


**Figure S5.** The effect of *Pten* silencing in synovial sarcoma development. (**A**) Western blotting shows *Pten* knockdown by shRNA. (**B**) Cumulative incidences of synovial sarcoma with shPten or negative control sh (ShNC). The number of mice used are indicated. Significance was examined by log-rank test.


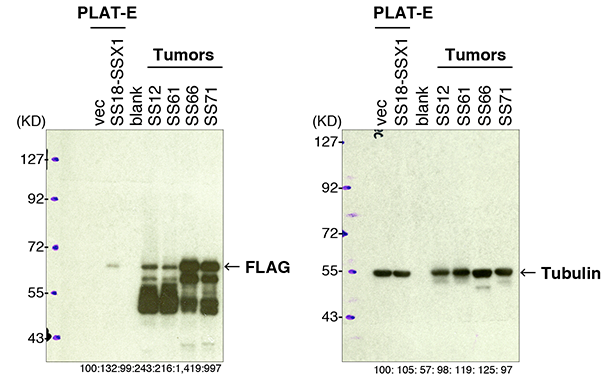


**Figure S6.** Original films of Figure 1D.


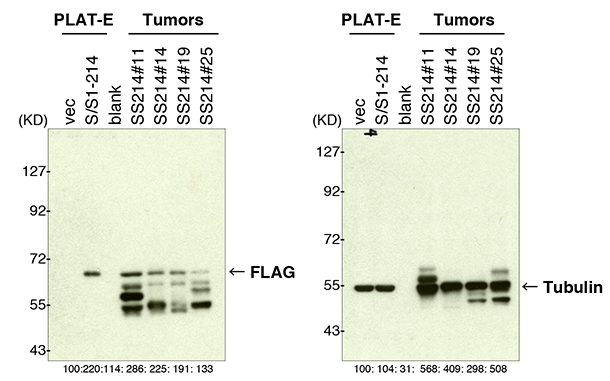


**Figure S7.** Original films of Figure 4E.


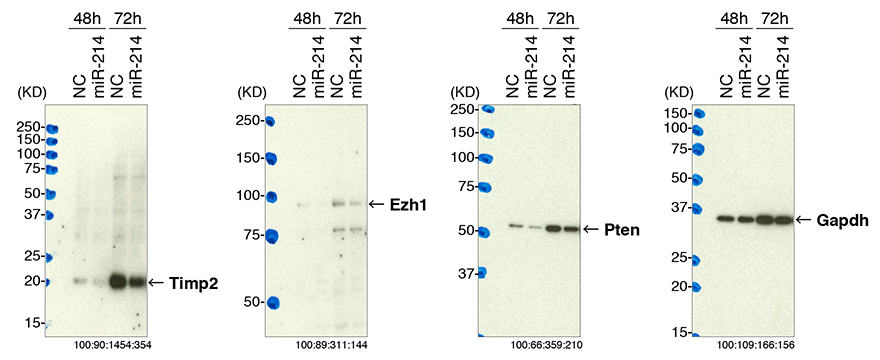


**Figure S8.** Original films of Figure 5D.

**Table S1–S5.**: Please check the accompanying MS Excel.

| 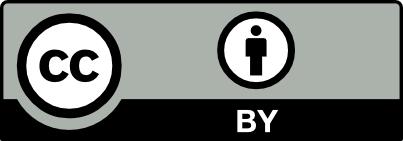 | © 2020 by the authors. Licensee MDPI, Basel, Switzerland. This article is an open access article distributed under the terms and conditions of the Creative Commons Attribution (CC BY) license (http://creativecommons.org/licenses/by/4.0/). |
| --- | --- |
